# Comparison of different therapeutic strategies for complete hydatidiform mole in women at least 40 years old: a retrospective cohort study

**DOI:** 10.1186/s12885-017-3749-8

**Published:** 2017-11-09

**Authors:** Peng Zhao, Qinqing Chen, Weiguo Lu

**Affiliations:** 1 Department of Gynaecology, Women’s hospital, School of Medicine, Zhejiang University, No.1 Xueshi Road, Hangzhou, Zhejiang Province China; 2 Department of Gynaecologic Oncology, Women’s hospital, School of Medicine, Zhejiang University, No.1 Xueshi Road, Hangzhou, Zhejiang Province China

**Keywords:** Complete hydatidiform mole, Gestational trophoblastic neoplasia, Gestational trophoblastic disease, Hysterectomy, Prophylactic chemotherapy, Uterine evacuation

## Abstract

**Background:**

There are three main therapeutic strategies, namely expectant management (dilation and curettage only), prophylactic chemotherapy and prophylactic total hysterectomy for treating older women with complete hydatidiform mole (CHM). However, the scientific community has so far, not unanimously accepted the above-mentioned methods. The objective of this study was to evaluate the effectiveness of these therapeutic strategies in preventing post-molar gestational trophoblastic neoplasia (GTN) pertaining to patients with CHM who were at least 40 years old.

**Methods:**

Hundred and seventy-one patients from our hospital who had histologically been diagnosed of CHM and underwent treatment from January 2004 to December 2013 were included. All patients were followed continuously for a minimum of 2 years after which relevant clinical data were extracted and analysed.

**Results:**

All patients were divided to three groups. Group 1 consisted of 124 patients, treated by expectant management, and the incidence of post-molar GTN was 37.1%. Group 2 included 12 patients who received prophylactic chemotherapy, with an incidence of 41.7%. The remaining 35 patients, Group 3, underwent prophylactic total hysterectomy, with the lowest incidence of 11.4%. A significantly lower incidence was noted in group 3 as compared to group 1 (*P* = 0.004). GTN patients who received prophylactic chemotherapy required, on average, longer time to be diagnosed of GTN and had higher probability of chemotherapy resistance (*P* = 0.031 and *P* = 0.024).

**Conclusions:**

This retrospective analysis showed that prophylactic total hysterectomy was the most effective therapeutic strategy for treating CHM in women at least 40 years old of age.

**Electronic supplementary material:**

The online version of this article (10.1186/s12885-017-3749-8) contains supplementary material, which is available to authorized users.

## Background

Gestational trophoblastic disease is a spectrum of interrelated diseases ranging from complete and partial hydatidiform mole to life-threatening gestational trophoblastic neoplasia (GTN), among which complete hydatidiform mole (CHM) is the most common form.

Despite the fact that CHM are mostly benign, risk of developing to GTN can reach up to 18-19% [1, 2]. Patients with CHM having at least one of the following characteristics are categorized as high risk: 1) Serum human chorionic gonadotropin (hCG) level prior to evacuation being greater than 100,000 IU/L. 2) An enlarged uterine size. 3) A theca lutein cyst with a diameter greater than 6 cm. 4) Maternal age being at least 40 years.

Increase in maternal age, which is the most frequently cited risk factor, has a proportional effect on the incidence of post-molar GTN [3, 4]. Tow, Savage and Tsukmoto et al. [5–7] reported that women greater than 40 years old had a probability of 23-37% of developing persistent GTN after uterine evacuation, while the likelihood in women older than 50 years old was as high as 31-56%. In comparison, relatively younger patients had a lower probability of 15-20% [8, 9].

Considering the age-incidence relationship involved, some centres might choose to offer prophylactic hysterectomy either instead of evacuation or following evacuation of a complete mole to reduce the risk of developing GTN in older patients who have completed their families. In addition, occasional centres may recommend prophylactic chemotherapy after molar evacuation as an alternative way to prevent post-molar GTN. However, these procedures are controversial. While some research [10–12] have found significant decrease in the probability of GTN transformation after undergoing prophylactic chemotherapy, other studies [13, 14], in turn, have found no significance in preventing post-molar GTN. The prophylactic effect of total hysterectomy is similarly controversial. While some studies demonstrated that it could potentially prevent the malignancy of CHM [15, 16, 14], others argued it could not reduce the incidence of GTN [17, 18]. Moreover, few of the above-mentioned studies specifically targeted patients who are at least 40 years old.

Due to the controversial aspect pertaining to the prophylactic effect of chemotherapy or hysterectomy in preventing GTN transformation and the lack of age-specific research, we conducted the current study to evaluate the effectiveness of three main therapeutic strategies, namely expectant management (dilation and curettage only), prophylactic chemotherapy and prophylactic total hysterectomy, in women with CHM who were at least 40 years old. The impact of these strategies on the remission of GTN was also thoroughly analysed.

## Methods

### Study population

Hundred and eighty-six patients with CHM, who were at least 40 years old and having undergone treatment in our hospital from January 2004 till December 2013, were shortlisted. Data including demographic characteristics, symptoms, operative records, laboratory data, pathological slides, imaging reports and common laboratory tests, were retrospectively reviewed.

Patients who met the following criteria were included in the study: 1) histologically confirmed diagnosis of CHM. 2) No evidence of local invasion or metastasis. 3) No evidence of residual trophoblastic tissue after surgical evacuation. 4) Aged at least 40 years old. All patients were followed for a minimum of 2 years. One patient was excluded due to incorrect diagnosis, 14 patients had incomplete follow-ups and were consequently, also excluded, leaving a cohort of 171 patients. The outcome, such as incidence of post-molar GTN, time interval to GTN diagnosis, number of courses of chemotherapy to cure, incidence of chemotherapy resistance (a plateau or rise in two consecutive hCG measurements after one course chemotherapy.), time required for hCG to normalize, were retrieved and analysed.

### Therapeutic strategies

Based on the protocol of our hospital, uterine evacuation (dilation and curettage, D & C) was conducted to all patients to confirm the diagnosis of CHM, after which three options were routinely offered: expectant management (observation without further treatment), prophylactic chemotherapy and prophylactic total hysterectomy. Ultrasound monitoring was conducted during molar evacuation to prevent uterine perforation and to ensure that there was no residual tissue in the uterus. The potential benefits and risks involved in each therapy were explicitly explained to patients and their responsible parties. After thorough considerations, a final decision was taken without the intervention of a medical professional.

Serum free beta hCG levels of patients who chose expectant management were monitored according to International Federation of Genecology and Obstetrics (FIGO) protocols. Patients who selected prophylactic chemotherapy were given methotrexate 0.4 mg/kg/d intravenously or intramuscularly for five consecutive days within 1 week after evacuation. We chose MTX as the first line regimen, and actinomycin as an alternate drug of choice in patients who were resistant to MTX, as both MTX and actinomycin were found to be effective (supplementary materials were included in the response letter). Prophylactic hysterectomy was performed within 1 week after evacuation by experienced surgeon on patients who selected total hysterectomy.

Based on the 2002 FIGO criteria [19] the diagnosis of post-molar GTN was maintained as follows: 1) an elevated hCG plateau (day 1, 7, 14 and 21); 2) rising hCG level (day 1, 7, 14); 3) an elevated hCG level for at least 6 months; 4) a histological diagnosis of choriocarcinoma.

An electrochemiluminescence kit was used to measure the level of free beta hCG in blood serum, using a sandwich-type detection method (Roche cobas®, Swiss).

### Statistical analysis

The data obtained were analysed using an independent *t* test, Pearson chi-square test or Fisher’s exact test, while Bonferroni correction was applied if multiple comparison tests were encountered. *P*-values of less than 0.05 were considered statistically significant. All calculations were conducted with SPSS (version 20.0) for Microsoft Windows.

## Results

### Demographic characteristics

All patients were 40-56 years old with a mean age of 46.9 years. Most of the patients were parous with a mean parity of 1.2 (range: 0-4), while mean gestational age was 9.5 weeks. 94.2% of the patients were diagnosed during first trimester and 5.8% during the second trimester. 60.9% of the patients had an initial hCG level of greater than 100,000 IU/L before evacuation, 24.4% had an enlarged uterine size over gestational age, while 13.3% had a theca lutein cyst greater than 6 cm. Three patients (1.75%) had a previous history of molar pregnancy. The demographic characteristics of the population studied were shown in Table 1.

**Table 1:** Demographic characteristics of the study population

Characteristic	Mean(SD)/rate	Range
Maternal age	46.9(3.8)	40-56
Gravidity	3.2(1.6)	0-11
Parity	1.2(0.5)	0-4
Gestational age(weeks)	9.5(3.0)	5-21
hCG level prior to evacuation greater than 100,000 IU/L	60.9%	–
Enlarged uterine size	24.4%	–
Theca lutein cyst greater than 6 cm	13.3%	–
Previous molar pregnancy	1.75%	

### Clinical presentation and physical signs

Abnormal vaginal bleeding accounted for 90.1% of all the manifestations. Abdominal pain and nausea with or without vomiting were less common, accounting 19.4 and 15.2% respectively, 8.6% of the patients presented with only amenorrhea as a manifestation. Each of fatigue, dizzy and abdominal distension occupied 2%. The least common clinical features were syncope and preeclampsia with a percentage of 1.0 and 0.9% respectively. The clinical presentation and physical signs are shown in Table 2.

**Table 2:** Clinical presentation and physical signs

Clinical presentation and physical signs	Rate
Abnormal vaginal bleeding	90.1%
Abdominal pain	19.4%
Nausea/vomiting	15.2%
Amenorrhea without symptoms	8.6%
Abdominal distension	2.0%
Fatigue	2.0%
Dizzy	2.0%
Syncope	1.0%
Preeclampsia	0.9%
Toxemia/hyperthyroidism/coagulopathy	0%

### The effectiveness of each therapeutic strategy

Table 3 shows the effectiveness of each therapeutic strategy in reducing post-molar GTN transformation. All patients were divided to three groups. Group 1 consisted of 124 patients, treated by dilation and curettage only (expectant management), and the incidence of post-molar GTN was 37.1%. Group 2 included 12 patients who received prophylactic chemotherapy, with an incidence of 41.7%. The remaining 35 patients, Group 3, underwent prophylactic total hysterectomy, with the lowest incidence of 11.4%. Pairwise comparisons were conducted with Bonferroni correction whereby significance was defined as *P* < 0.017 (0.05/3). There was a significant difference in the general distribution of GTN (χ^2^ = 8.777, *P* = 0.012). A significantly lower incidence of post-molar GTN was noted in group 3 (*P* = 0.004 as compared to group 1). It is worth mentioning that the incidence of post-molar GTN between group 1 and group 2 as well as between group 2 and group 3 showed no significant differences. (*P* = 0.763 and *P* = 0.035, respectively). A comparison of the clinical characteristics such as maternal age, gestational age, gravidity, parity, pre-evacuation hCG level, uterine size and a theca lutein cyst of each group was conducted and no statistical significance were found (Additional file 1: Table S1-S3).

**Table 3:** Effect of different therapeutic strategies in reducing post-molar GTN

Therapeutic strategies	N	No. of GTN	Incidence of post-molar GTN
Group 1	124	46	37.1%
Group 2	12	5	41.7%
Group 3	35	4	11.4%

Fisher’s exact test: Group 1 VS Group 2, P = 0.763; Group 2 VS Group 3, *P* = 0.035. Group 1 VS Group 3, χ^2^ = 8.342, P = 0.004; Significance was defined as P < 0.017 (0.05/3) for Bonferroni correction

Table 4 shows the comparison of the various characteristics of patients who received hysterectomy. No significant differences were noted between remission group and GTN group in clinical features such as maternal age, gravity, parity, gestational age, pre-evacuation hCG level greater than 100,000 IU/L, enlarged uterine size and a theca lutein cyst greater than 6 cm.

**Table 4:** Comparison of characteristics of the patients who received hysterectomy

Characteristic	GTN	Remission	P-value
Maternal age (year)	47.7 ± 3.1	47.3 ± 5.6	0.816
Gravidity	3.6 ± 2.1	2.7 ± 0.5	0.437
Parity	1.2 ± 0.5	1.3 ± 0.5	0.848
Gestational age (weeks)	10.3 ± 3.9	12.5 ± 6.4	0.338
hCG level prior to evacuation greater than 100,000 IU/L	50%	74.2%	0.561
Enlarged uterine size	0%	35.5%	0.285
Theca lutein cyst greater than 6 cm	0%	16.1%	1.0

### The influence of prophylactic chemotherapy

Patients with GTN were divided into three groups based on their primary therapeutic strategies after molar evacuation. Expectant group was defined as the group of patients who received no further treatment after D & C (group I). Prophylactic chemotherapy grouped patients who received prophylactic chemotherapy (group II). Hysterectomy group comprised of patients who received prophylactic total hysterectomy (group III). The outcome of patients with GTN was shown in Table 5. Significant differences were noted in time interval to GTN diagnosis and incidence of chemotherapy resistance (*P* = 0.031 and *P* = 0.024, respectively, group I versus group II). No statistically significances were found in the traditional risk factors or prognostic scores (Additional file 2: Table S4-S5).

**Table 5:** The outcome of patients with GTN

Group of patients with GTN	Time to GTN diagnosis (day)	No. of courses of chemotherapy to cure	Chemotherapy resistance	Time for hCG to remission (day)	Follow-up
Group I (*n* = 46)	54.0 ± 38.5	3.2 ± 1.9	23.7%(9/38)	59.6 ± 26.4	46 remission, 1 recurred 4 years later and died of brain metastasis
Group II (*n* = 5)	94.6 ± 35.9^*^	4.2 ± 2.4	80.0%(4/5)^**^	60.4 ± 18.6	5 remission,0 death
Group III (n = 4)	60.7 ± 43.0	2.7 ± 1.5	25.0%(1/4)	52.0 ± 23.3	4 remission,0 death

^*^ P = 0.031, compared to Group I; ^**^ P = 0.024 compared to Group I

Risk factors such as pre-evacuation hCG level, a theca luteal cyst over 6 cm and an enlarged uterus over presentation date and prognostic scores were evaluated among groups and no statistically significances were found

## Discussion

This retrospective study found that prophylactic total hysterectomy significantly decreased the incidence of post-molar GTN in women with CHM who are at least 40 years old. Prophylactic chemotherapy had no effect in preventing GTN for older patients, on the contrary, it increased time interval to GTN diagnosis and the incidence of chemotherapy resistance.

D & C is the preferred treatment option for young patients with CHM, while for older patients who are at least 40 years old and no longer require fertility, total hysterectomy or prophylactic chemotherapy might be considered due to the higher incidence of post-molar GTN. However, there is no general consensus on the two above-mentioned therapeutic strategies. It can be stated that each method has its advantages and disadvantages. The objective in managing CHM lies in preventing post-molar GTN which subsequently leads to metastasis. Therefore, any therapeutic strategy should be weighed on the basis of prevention. Consequently, the present study was conducted to assess the effectiveness of the three main therapeutic strategies based on the incidence of post-molar GTN.

This study predominantly shows that prophylactic total hysterectomy significantly decreased the incidence of post-molar GTN, indicating that total hysterectomy might be the best therapeutic strategy for older patients who had completed childbearing. Hysterectomy was first introduced in 1966 [20]. Since then, concerns have been raised upon the potential vascular dissemination of trophoblastic tissue due to the surgical procedure involved [2, 21–23]. Meanwhile, the liberal use of hysterectomy in the elderly and multiparous patients has also been favoured [7, 15, 20, 16, 14]. Several authors [2, 15] claimed a 10-20% chance of malignancy in hysterectomy group compared to 33.3% of non-hysterectomy group. However, considering the above mentioned studies including patients across all ages and selecting both types of hydatidiform mole, the results are not applicable to older patients with CHM who are at least 40 years old. Sporadic study [16, 14] aimed for this age-specific group of patients showed that hysterectomy might result in a better outcome, however, due to the marginal number of patients receiving hysterectomy (only 6 patients), the clinical significance is highly doubted. In comparison, our study consisted of 37 patients in the prophylactic hysterectomy group, which theoretically should lead to more reliable results. It is worth mentioning that a recent study [17] showed that hysterectomy after 40 years old in women with HM does not reduce the incidence of GTN. In this study, a total of 76 patients with HM who were over 40 years old were included. The incidence of post-molar GTN were 58.3 and 29.7%, respectively (total hysterectomy versus uterine evacuation, *P* = 0.094). The authors concluded that primary hysterectomy might not be able to prevent post-molar GTN. However, they included patients who were diagnosed with invasive HM in the total hysterectomy group, which could increase the probability of post-molar GTN. We deemed that this selection bias could seriously weaken the confidence of the results and lead to incorrect conclusion.

Another major finding of the current study is that prophylactic chemotherapy had a similar incidence of post-molar GTN as the expectant group. The use of prophylactic chemotherapy has been based on the assumption that the development of GTN is pre-determined, adding to the fact that metastatic GTN spreads via the bloodstream and that high serum level of cytotoxic agents around the time of evacuation should reduce the ability of the trophoblastic cells to invade or metastasize [24]. However, prophylactic chemotherapy exposed 80% of patients unnecessarily to toxic side effects [25] and might lead to incomplete protection against persistent tumour [24]. Previous studies [9, 26] reported a significant reduction in the rate of GTN transformation in adolescents and adults, while the targeted group was mostly below 40 years old, whether prophylactic chemotherapy could prevent post-molar GTN in women above 40 years old is still unclear. Our study was the first of its kind to study patients who were at least 40 years old and found that prophylactic chemotherapy could not reduce the incidence of post-molar GTN. In addition, we found that prophylactic chemotherapy increased time interval to GTN diagnosis and the incidence of chemotherapy resistance. Therefore, it can be assumed that prophylactic chemotherapy delayed the diagnosis of GTN thus lead to chemotherapy resistance, which was also observed by a Cochrane systematic review [27]. It should be noted that the outcome of prophylactic chemotherapy in our study was based on the MTX 5-day regimen, therefore, it should be carefully interpreted as there are alternate regimens which may lead to better outcomes. Some may question the validity of our results as our study showed an opposite outcome in prophylactic chemotherapy compared to published randomized controlled trials (RCTs) [8, 28, 29]. However, the major difference between our study and RCTs is the target group, that is, we recruited patients who were being at least 40 years old with or without other high risk factors, which may lead to different results. Therefore, despite the fact that there is divergence, we strongly believe that the results of our study are reliable because we aimed at investigating an age specific group of patients who are 40 years old or above.

Maternal age, gestational age, hCG level prior to evacuation greater than 100,000 IU/L, uterine enlargement, a theca lutein cyst greater than 6 cm have been frequently reported to be associated with GTN. In our study, such clinical characteristics were evaluated and no significant differences were noted. We also investigated the clinical presentation of the study population; few were associated with toxemia, hyperthyroidism or coagulopathy, which is inconsistent with previous studies [22, 30]. This phenomenon can be explained by the earlier diagnosis with the improvement of technology [31–33], especially the world-wide application of ultrasonography. As in our study, 94.2% of the patients were diagnosed in the first trimester when the symptoms of toxemia, hyperthyroidism or coagulopathy are usually rare.

The most common clinical manifestation was abnormal vaginal bleeding, a few accompanied with abdominal pain, nausea or vomiting, which is consistent with the clinical manifestations of young patients. Interestingly, 8.6% of patients in our study presented with amenorrhea as the only symptom, compared to just 0.8% in young patients [34], hinting a major difference in presentation. This phenomenon should alert physicians during clinical practice. Since older patients were easily neglected and misdiagnosed with amenorrhea due to the perimenopausal period around 40s, pregnancy might be suspected and abortion would be performed without pre-caution, exposing patients to uterine perforation and heavy bleeding. This study emphasizes on the fact that appropriate diagnostic attention should be given to older patients requesting for elective termination of pregnancy presenting with no complaints, and that, simultaneously, all products of conception after evacuation should be pathologically reviewed to exclude hydatidiform mole.

The limitation of this study lies on its retrospective aspect. Unknown bias might have been introduced because selection of the therapeutic strategies was not based on random. Limited patients were chosen for this study, implying that it is impracticable to perform subgroup analysis and identify bias. For instance, in order to detect selection bias, we compared the traditional risk factors and clinical characteristics between each group and some of the comparisons are unable to conduct due to the marginal number of participants. Moreover, the limited number of participants in prophylactic chemotherapy arm may affect the results.

The strengths of our study are that, compared to previous studies, we recruited larger size of patients, used explicit criteria and improved the study design. In the light of our knowledge, it is one of the largest retrospective cohorts reported in literature pertaining to women with complete hydatidiform mole who are at least 40 years old.

## Conclusion

We conclude that prophylactic total hysterectomy is beneficial in patients with CHM who are at least 40 years old. This study therefore, proposes total hysterectomy be considered as the optimal treatment option for women over 40 who have completed their family. This is because it appears to significantly reduce the risk of subsequent GTN compared to expectant management. However, further larger studies are required to substantiate our finding. Finally, in agreement with prior work, our data do not support the use of prophylactic chemotherapy.

## Additional files

Additional file 1: Table S1-S3. Comparisons were conducted among groups based on different therapeutic strategies and no significant differences were found. Comparison of clinical characteristics between prophylactic chemotherapy group and expectant group was presented in Table S1. Comparison of clinical characteristics between expectant group and hysterectomy group was presented in Table S2. Comparison of clinical characteristics between prophylactic chemotherapy group and hysterectomy group was presented in Table S3. (DOCX 20 kb)

Additional file 2: Table S4-S5. Comparisons were conducted between groups of patients with GTN based on therapeutic strategies and no significant differences were noted. Comparison of clinical characteristics between prophylactic chemotherapy group and expectant group was presented in Table S4. Comparison of clinical characteristics between hysterectomy group and expectant group was presented in Table S5. (DOCX 19 kb)

## Abbreviations

CHM: Complete hydatidiform mole
D & C: Dilation and curettage
FIGO: International Federation of Genecology and Obstetrics
GTN: Gestational trophoblastic neoplasia.
hCG: human chorionic gonadotropin.
