# Supplementary material for: Comparison of different therapeutic strategies for complete hydatidiform mole in women at least 40 years old: a retrospective cohort study
Source: BMC Cancer. 2017 Nov 9;17:733. doi: 10.1186/s12885-017-3749-8 (PMC5679144; doi:10.1186/s12885-017-3749-8)
Supplement: Supplementary file 1 — Comparisons were conducted among groups based on different therapeutic strategies and no significant differences were found. Comparison of clinical characteristics between prophylactic chemotherapy group and expectant group was presented in Table S1. Comparison of clinical characteristics between expectant group and hysterectomy group was presented in Table S2. Comparison of clinical characteristics between prophylactic chemotherapy group and hysterectomy group was presented in Table S3. (DOCX 20 kb) [file 12885_2017_3749_MOESM1_ESM.docx]

**Comparison among groups based on different therapeutic strategies** (expectant, prophylactic chemotherapy or hysterectomy)

Table S1. Comparison of clinical characteristics between prophylactic chemotherapy group and expectant group

| Characteristic | expectant | prophylactic chemotherapy | P-value |
| --- | --- | --- | --- |
| Maternal age (year) | 46.6±3.8 | 46.9±4.1 | 0.805 |
| Gravidity | 3.2±1.5 | 2.9±1.4 | 0.547 |
| Parity | 1.2±0.6 | 1.2±0.4 | 0.855 |
| Gestational age (week) | 9.2±2.3 | 9.2±2.6 | 0.955 |
| hCG level prior to evacuation  over 100000IU/L | 87.2% | 80% | 0.177 |
| Enlarged uterine size | 14.9% | 42.9% | 0.109 |
| Theca lutein cyst over 6cm | 12.5% | 12.5% | 1.000 |

Table S2. Comparison of clinical characteristics between expectant group and hysterectomy group

| Characteristic | expectant | hysterectomy | P-value |
| --- | --- | --- | --- |
| Maternal age (year) | 46.6±3.8 | 47.6±3.4 | 0.163 |
| Gravidity | 3.2±1.5 | 3.5±2.0 | 0.343 |
| Parity | 1.2±0.6 | 1.2±0.5 | 0.942 |
| Gestational age (week) | 9.2±2.3 | 10.7±4.3 | 0.065 |
| hCG level prior to evacuation  over 100000IU/L | 87.2% | 72.4% | 0.120 |
| Enlarged uterine size | 14.9% | 34.6% | 0.076 |
| Theca lutein cyst over 6cm | 12.5% | 14.8% | 1.000 |

Table S3. Comparison of clinical characteristics between prophylactic chemotherapy group and hysterectomy group

| Characteristic | prophylactic chemotherapy | hysterectomy | P-value |
| --- | --- | --- | --- |
| Maternal age (year) | 46.9±4.1 | 47.6±3.4 | 0.554 |
| Gravidity | 2.9±1.4 | 3.5±2.0 | 0.363 |
| Parity | 1.2±0.4 | 1.2±0.5 | 0.800 |
| Gestational age (week) | 9.2±2.6 | 10.7±4.3 | 0.260 |
| hCG level prior to evacuation  over 100000IU/L | 80% | 72.4% | 1.000 |
| Enlarged uterine size | 42.9% | 34.6% | 0.350 |
| Theca lutein cyst over 6cm | 12.5% | 14.8% | 1.000 |
