# Supplementary material for: Comparison of different therapeutic strategies for complete hydatidiform mole in women at least 40 years old: a retrospective cohort study
Source: BMC Cancer. 2017 Nov 9;17:733. doi: 10.1186/s12885-017-3749-8 (PMC5679144; doi:10.1186/s12885-017-3749-8)
Supplement: Supplementary file 2 — Comparisons were conducted between groups of patients with GTN based on therapeutic strategies and no significant differences were noted. Comparison of clinical characteristics between prophylactic chemotherapy group and expectant group was presented in Table S4. Comparison of clinical characteristics between hysterectomy group and expectant group was presented in Table S5. (DOCX 19 kb) [file 12885_2017_3749_MOESM2_ESM.docx]

**Comparison among groups of patients with GTN**

Table S4. Comparison of clinical characteristics between prophylactic chemotherapy group and expectant group

| Characteristic | expectant | prophylactic chemotherapy | P-value |
| --- | --- | --- | --- |
| Maternal age (year) | 47.4±3.7 | 44.2±2.9 | 0.070 |
| Gravidity | 3.7±2.1 | 3.2±1.3 | 0.636 |
| Parity | 1.2±0.7 | 1.2±0.4 | 0.888 |
| Gestational age (week) | 8.7±2.2 | 9.1±2.7 | 0.745 |
| hCG level prior to evacuation  over 100000IU/L | 63.3% | 100% | 0.290 |
| Enlarged uterine size | 40% | 100% | 0.455 |
| Theca lutein cyst over 6cm | 0% | 0% | NA |
| High risk | 4.8% | 0% | 1.000 |

Table S5. Comparison of clinical characteristics between hysterectomy group and expectant group

| Characteristic | expectant | hysterectomy | P-value |
| --- | --- | --- | --- |
| Maternal age (year) | 47.4±3.7 | 47.3±5.6 | 0.070 |
| Gravidity | 3.7±2.1 | 2.8±0.5 | 0.636 |
| Parity | 1.2±0.7 | 1.3±0.5 | 0.888 |
| Gestational age (week) | 8.7±2.2 | 17.1±6.1 | 0.298 |
| hCG level prior to evacuation  over 100000IU/L | 63.3% | 50% | 1.000 |
| Enlarged uterine size | 40% | 0% | 1.000 |
| Theca lutein cyst over 6cm | 0% | 0% | NA |
| High risk | 4.8% | 0% | 1.000 |
